# Supplementary figures and images for: Kynurenic acid, a key L-tryptophan-derived metabolite, protects the heart from an ischemic damage
Source: PLoS One. 2023 Aug 24;18(8):e0275550. doi: 10.1371/journal.pone.0275550 (PMC10449225; doi:10.1371/journal.pone.0275550)

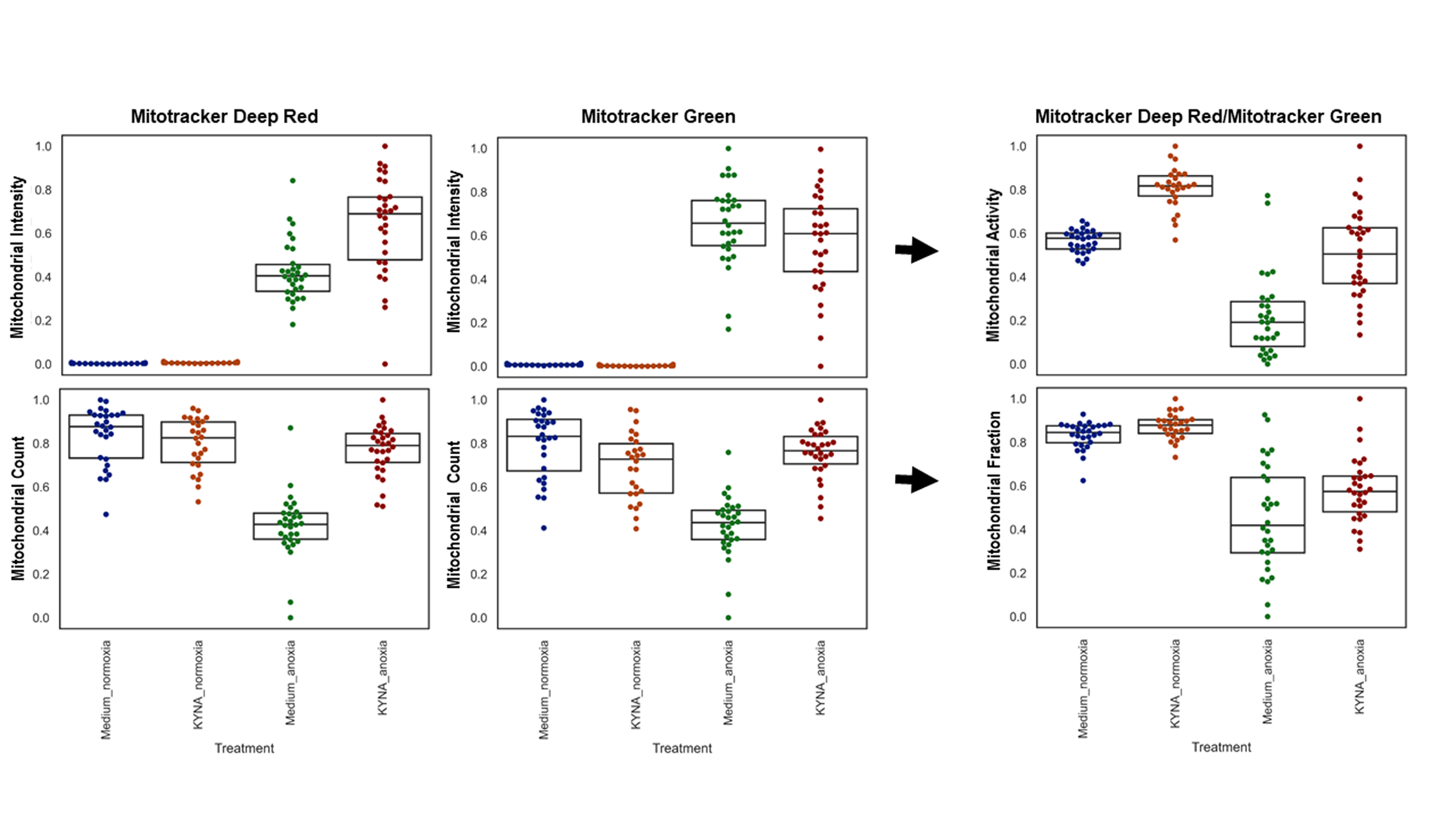

Supplement: S1 Fig — (A) Quantification of Mito Activity and Mito fraction as documented in Fig 4. (B) Box plots graph representation of MitoTracker Deep Red intensity, MitoTracker red intensity, and cell intensity. (C) Box plots graph re-presentation of mitochondria elongation and area (24–30 replicates per arm). (TIF) [file pone.0275550.s001.tif]

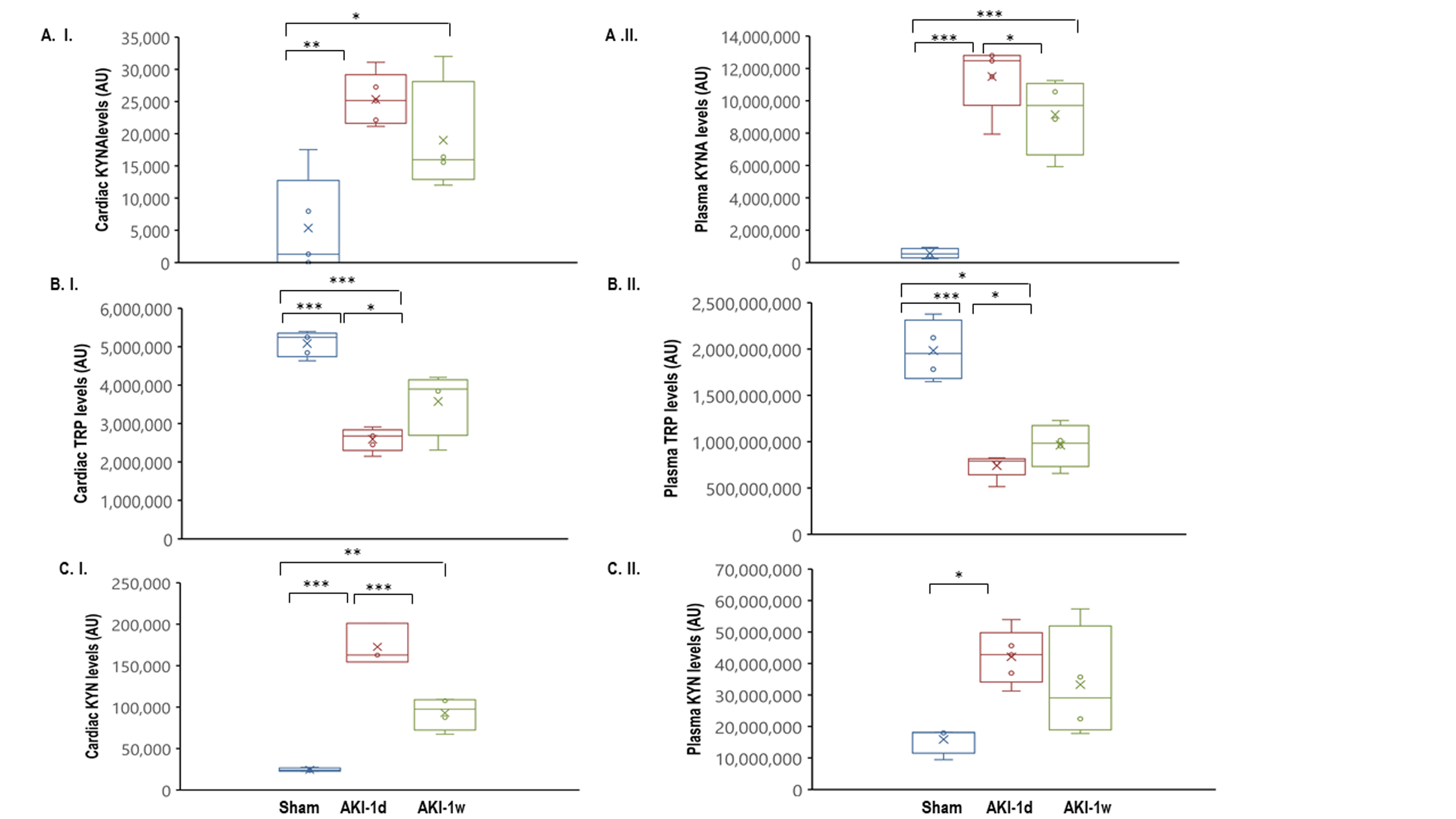

Supplement: S2 Fig — (TIF) [file pone.0275550.s002.tif]
