# Supplementary material for: Kynurenic acid, a key L-tryptophan-derived metabolite, protects the heart from an ischemic damage
Source: PLoS One. 2023 Aug 24;18(8):e0275550. doi: 10.1371/journal.pone.0275550 (PMC10449225; doi:10.1371/journal.pone.0275550)
Supplement: Attachment — Submitted filename: Revision letter 23022023.docx [file pone.0275550.s005.docx]

February, 2023

To: Dr. Daniel M. Johnson, Academic editor

**Re: Revision to manuscript PONE-D-22-25974:**

Dear Dr. Johnson,

We would like to thank the reviewers and the editor for the in‑depth analysis of our work and for raising several important points that needed clarification. We appreciate the time and effort expended on our behalf. We have made significant modifications to the manuscript accordingly, and out point-by-point responses are listed below.

We hope that our revised manuscript will now be found suitable for publication in PLOS ONE.

Sincerely,

Dr. Michal Entin-Meer and Prof. Gad Keren

**General comments:**

1. Please ensure that your manuscript meets PLOS ONE's style requirements, including those for file naming. The PLOS ONE style templates can be found at 
<https://journals.plos.org/plosone/s/file?id=wjVg/PLOSOne_formatting_sample_main_body.pdf> and 
<https://journals.plos.org/plosone/s/file?id=ba62/PLOSOne_formatting_sample_title_authors_affiliations.pdf-> **Done**.

2. We note that the grant information you provided in the ‘Funding Information’ and ‘Financial Disclosure’ sections do not match.

When you resubmit, please ensure that you provide the correct grant numbers for the awards you received for your study in the ‘Funding Information’ section. **Done.**

3. We note that you have included the phrase “data not shown” in your manuscript. Unfortunately, this does not meet our data sharing requirements. PLOS does not permit references to inaccessible data. We require that authors provide all relevant data within the paper, Supporting Information files, or in an acceptable, public repository. Please add a citation to support this phrase or upload the data that corresponds with these findings to a stable repository (such as Figshare or Dryad) and provide and URLs, DOIs, or accession numbers that may be used to access these data. Or, if the data are not a core part of the research being presented in your study, we ask that you remove the phrase that refers to these data. **Corrected**

**Reviewer 1:**

1. **Page 6, Line 119: Why 6 mice were excluded due to “normal cardiac function” on Day 1 after LAD ligation? Was this a surgical technical issue or due to resistance of female mice to myocardial ischemia? If so, why the authors chose female mice not male rats for AMI studies, considering the AKI studies were performed in male rats? Why used different animal species and genders?**

"Normal cardiac function" on day 1 post AMI induction means that the LAD ligation was technically unsuccessful (i.e., MI was not induced in these animals), therefore these animals were excluded from the experiment. In order to clarify the issue, we modified this sentence under "Materials & Methods" which now reads: Five animals from the control group and six animals from the experimental group were excluded from the analysis due to one of the following reasons: normal cardiac function according to echocardiography applied on day 1, suggesting that LAD ligation was unsuccessful (6 animals), animal's death prior to endpoint (4 animals) or weight loss of > 15% (1 animal) (lines 119-123).

The strain and gender for each experimental procedure (AKI and AMI) was chosen based on practical reasons *per se*. We added an explanation as follows: "Male Lewis rats (250–350 gr)/ 12-week old female BALB/C mice were purchased from Envigo, Israel. Male Lewis rats were used for AKI studies, as previously established in our laboratory for 5/6 nephrectomy models (9). Female mice were utilized in the AMI studies, in accordance with the extensive experience gathered in our laboratory (15, 16) and the lower cardiac rupture rate of female compared to male mice following AMI induction (17) (lines 95-99). We agree that this issue may be confusing, therefore we also added the following sentence under "Study limitation: "Likewise, the beneficial effect of KYNA should also be validated in an AMI model in male rats, in line with the *in vitro* beneficial effect demonstrated herein for anoxic H9C2 rat myocytes and in view of the entire metabolomics analysis that was conducted in a male rat AKI model" (lines 456-459).

2. **Page 6, Line 130 (currently 134): Please correct “into 5 µM slices” with “into 5 µm slices**”-done.
3. **Page 7, Line 146 to 154: The description of statistical analysis should be removed here and merged to Page 9, Line 189 to 195, in order to avoid redundancy**

Corrected. The explanation regarding the statistical analysis is currently merged (lines 184-196).

4. **Page 9, Line 165 and 171: How the sole concentration of 10 mM kynurenic acid was selected for the H9C2 experiments? Is 10 mM a physiologically achievable concentration that reflects tryptophan degradation in vivo? Please justify.**

As the reviewer noted, when reading the "Materials & Methods" section which precedes the Results section, it is unclear why 10 mM dose of KYNA was used in the *in vitro* experiments. Therefore, we omitted the dose from "Materials and Methods", while further clarifying the issue in the Results as explained in lines 266-266: “In view of the observed dramatic effect of KYNA when given at 10 mM concentration, we chose to use this dose in all our subsequent *in vitro* experiments”, as also shown in Figure 3A. The 10 mM dose was then mentioned in the description of the subsequent in vitro experiments and the observed results (lines: 267, 274, 287).

5. **In Discussion section, it would be helpful to refer the particular figure(s) or table(s) to justify each of your claims or reasoning, based on the results of the current study**- Done.
6. **Page 19, Line 427-428: The paragraph of “Study Limitation” seems to be quite superficial and needs to be expanded with further details and/or explanations. For example, why did the authors use two different species and genders, male rats versus female mice in AKI and AMI studies? Also mitochondrial study was done only H9C2 cells, which have different cellular characteristics from primary cardiomyocytes. This should be discussed as a study limitation.**

We thank the reviewer for this important comment. The suggested remarks were added to "Study limitations (lines 456-465).

7. **Do you think the release of cardioprotective substances to circulation after AKI may potentially explain the phenomenon of “remote preconditioning”? If so, please add some discussion on this possibility.**

We thank the reviewer for raising this important issue. We indeed believe that the excess release of KYNA to the plasma following AKI may represent (at least in part) remote ischemic preconditioning. A newly-released Science paper as well as additional recent study point to this phenomenon. We added the data to the Discussion: "In this respect it is worthwhile to note innovative observations suggesting that plasma KYNA may promote remote ischemic preconditioning by binding to GPR35 expressed on cardiomyocytes (45). GPR35 bound to KYNA can bind to the mitochondrial protein ATP synthase inhibitory factor subunit 1 (ATP1F1), thus regulating the ATP synthase complex, leading to its subsequent dimerization (44). The activation of ATP synthase potentially overcomes the overall loss of ATP levels, which contributes to cell death upon ischemia. In addition, it has recently been reported that KYNA may enable an enhanced recovery and functionality of an injured ischemic/reperfusion aorta, potentially by promoting reactive oxygen (ROS)- dependent tissue repair via acting on oxidant enzymes (46)"(lines 407-415).

8. **Page 25, Ref. 42: The journal volume/page numbers are missing for this reference**- corrected.
9. **Figure 6 should use scatter dot plots, instead of bar graphs, in order to reveal all individual data points. Also please report the heart rate data for all groups of mice, since previous study (Ref. 22) showed that in vivo treatment of kynurenic acid significantly reduced heart rate in rats. Please discuss if such heart rate reduction contribute to the cardioprotective effects of kynurenic acid in the present study.**

We rearranged Figure 6 so that it currently uses box plots (instead of bar graphs).

The heart rate in all echocardiographic analyses was set to 400-500 bpm, prior to taking the measurements. Due to the isoflurane anesthesia and the chest pressure inflicted by the catheter, we could not achieve a heart rate of over 500 bpm. However, a heart rate between 400-500 bpm is actually the standard of our echocardiography measurements, as presented in our earlier PLOS ONE papers (Entin-Meer et al, 2017, Bigelman et *al*, 2018, Cohen et al, 2020). It is also considered an acceptable rate according to Lindsey *et al.*, Am J Physiol Heart Circ Physiol, 2018. We added this clarification under "Material and Methods-Echocardiography" as follows: "Cardiac parameters on day one and day 30 post-infarction were obtained by echocardiography (Vevo 2100, VisualSonics, Toronto, Canada), while heart rate was kept between 400-500 bpm in accordance with the guidelines for echocardiographic measurements in the murine heart (19)" (lines 125-128).

**Reviewer 2:**

1. **In Figure 1, the authors used 26 differentially-expressed cardiac and plasma metabolites at both time points: 1 day and 1 week relative to sham. However, the reviewer cannot understand the reason underlying the time point selection.**

We chose two time points in AKI- 1d and 7d, which may represent potentially-transient vs potentially-persistent AKI. We added this explanation in lines 112-113 ("Materials & Methods"). Only the metabolites which were modified at these two time points relative to control were considered significant in our metabolomics analysis (Figure 1, Figure 2).

2. **It requires to further evaluate the concentration of KYNA in the tissue and serum.**

We added Fig S4 (supplementary material) showing the raw data obtained for KYNA, KYN and TRP in the global metabolomics analysis (AU). As expected according to accumulated published data, we could see that the metabolites' levels both in the blood and the cardiac sections are as follows: TRP> KYN> KYNA. We added the following explanation to the beginning of the Discussion (lines 366-371): "In line with the known TRP metabolism pathway and the reported data from human and rodents (29, 31), our analysis demonstrates that in both cardiac and plasma samples the metabolites' levels are: TRP > KYN> KYNA, so that TRP levels are higher than KYNA by at least 3-order of magnitude (S4 Fig). Moreover, it has been suggested that in CKD patients the plasma levels of TRP decrease from 96.3± 18.3 to 62.9 ± 22.8 μmole/L, while KYNA levels are increased from 24.2± 7.8 to 43.3 ± 18.2 nmole/L; p< 0.001) (29). Yet, we agree that the actual concentration of KYNA should be evaluated via targeted-metabolimcs analysis (as opposed to the global analysis presented in the current manuscript). We added the relevant statement to "Study limitation" as follows: "In addition, future studies should include measurements of the actual KYNA levels in the heart and plasma via targeted- metabolomics analysis" (lines 463-465).

3. **Although the authors reported that KYNA is able to reduce ischemia-mediated H9C2 damage, the molecular mechanisms underlying cell death has not been addressed.**

In accordance with a recently-published Science paper (Wyant et al, Mitochondrial remodeling and ischemic protection by G protein-coupled receptor 35 agonists), as well as by Lima et al, 2021- we added a paragraph to the discussion that describes the potential molecular/intracellular mechanisms underlying the cell death rescue by KYNA (lines 407-415): " In this respect it is worthwhile to note innovative observations suggesting that plasma KYNA may promote remote ischemic preconditioning by binding to GPR35 expressed on cardiomyocytes (45). GPR35 bound to KYNA can bind to the mitochondrial protein ATP synthase inhibitory factor subunit 1 (ATP1F1), thus regulating the ATP synthase complex leading to its subsequent dimerization. The activation of ATP synthase potentially overcomes the overall loss of ATP levels, which contributes to cell death upon ischemia. In addition, it has recently been reported that KYNA may enable an enhanced recovery and functionality of an injured ischemic/reperfusion aorta, potentially by promoting reactive oxygen (ROS)- dependent tissue repair via acting on oxidant enzymes (46)" In addition, we have demonstrated that the ischemia-mediated cell-death salvaged by KYNA is partly achieved by reducing the oxidative stress (Figure 5).

4. **The 5/6 nephrectomy is actually a chronic renal failure model but not acute kidney injury.**

We agree that 5/6 nephrectomy is a known model for CKD. Yet, the metabolomics analysis performed on the plasma samples of the experimental animals revealed a significant increase in both creatinine and urea levels relative to sham-operated control animals, implying to renal dysfunction occurring as early as 1d post model induction. We added box plots showing these data to Figure 1 (Fig1A) and a paragraph regarding model validation in the beginning of the Results section (lines 202-209).

5. **Acute hypoxia exposure cannot simulate the model of cardiac injury after AKI.**

We agree. Nevertheless, since AMI is an unfortunate prevalent phenomenon following renal injury, we thought to assess the potential effect of KYNA should an ischemic event occurs. We added this statement on line 334-335 as follows: "Based on the *in vitro* data pointing to cardiac protection by KYNA upon O_2_ restriction, and due to the known significantly increased risk for cardiac ischemic events in patients suffering from renal failure (28), we sought to assess the effects of KYNA in an *in vivo* model for AMI induced by a permanent LAD ligation." In addition, it is worthwhile to mention that an exposure of cardiac cells to anoxic/hypoxic conditions was already utilized by our group to simulate cardiac infarction (though obviously not necessarily due to AKI) as reported in Entin-Meer et al, PLoS One, 2014.

We hope that you will find the corrected manuscript entitled: "Kynurenic acid, a key L-tryptophan-derived metabolite, protects the heart from an ischemic damage" interesting for the readers of PLoS ONE.

Sincerely,

Dr. Michal Entin-Meer
