# Supplementary material for: Kynurenic acid, a key L-tryptophan-derived metabolite, protects the heart from an ischemic damage
Source: PLoS One. 2023 Aug 24;18(8):e0275550. doi: 10.1371/journal.pone.0275550 (PMC10449225; doi:10.1371/journal.pone.0275550)
Supplement: Attachment — Submitted filename: Revision letter 16052023.docx [file pone.0275550.s006.docx]

May 15^th^, 2023

To: Dr. Daniel M. Johnson, Academic editor

**Re: Revision to manuscript PONE-D-22-25974R1:**

Dear Dr. Johnson,

We would like to thank the reviewers and the editor for the in‑depth analysis of our work and for raising an additional point that needed clarification. We appreciate the time and effort expended on our behalf. We have modified to the manuscript accordingly, as listed below.

**Reviewer #3:**  My main concern relates to the use of MitoTracker Green for mitochondrial structure and MitoTracker Deep Red for mitochondrial function (page 13, line 288-289). How do the authors justify this? MitoTracker fluorescent probes are useful for mitochondria localization. Also, an increase in superoxide production in anoxia, as the increase reported in H9C2 cells, seems counterintuitive, since superoxide is generated from oxygen. How did the authors achieved anoxia, i.e. 0% oxygen

**Response:** We thank the reviewer for raising this issue. We agree that the term "Mitochondrial structure" with respect to MitoTracker Green is not accurate and should be changed to "Mitochondrial content/mass". Yet, even though fluorescent MitoTracker probes are successfully utilized for mitochondrial localization/co-localization experiments as the reviewer rightfully noted, they could also be applied as a useful tool in evaluating mitophagy (MitoTracker Green which selectively accumulates at the mitochondrial matrix) and alterations in the mitochondrial membrane potential (MitoTracker Red whose levels of accumulation in the mitochondria depend on the membrane potential) in live cells (added Ref 24-26).

As for the MitoSOX staining: it is reported to successfully detect mitochondrial ROS production due to oxygen deprivation in various cells including cardiomyocytes, as previously reported (added Ref 27, 28).

In view of the above-mentioned:

1. We changed the term "Mitochondrial structure" to "Mitochondrial content" or "Mitochondrial mass" when discussing the data obtained with MitoTracker Green.
2. We added several references to justify the use of the MitoTracker dyes for the assessment of mitochondrial content and activity (Ref 24-26) as well as the use of MitoSOX dye for the assessment of ROS production (Ref 27, 28) in live H9C2 cells following oxygen deprivation.
3. We added clarification regarding the utilization of the dyes to "Materials & Methods (lines 175-179) and "Results" (lines 291-292 and 315-317). We also added the following sentences under Discussion (lines 422-427): "The data presented herein demonstrate that KYNA somewhat decreased the cellular viability, induced some degradation of the mitochondria, reduced the mitochondrial membrane potential and induced marginal oxidative damage under physiological conditions, as indicated (Fig 3-5), in line with other reports (52). Interestingly, however, under anoxic conditions, KYNA significantly increased the viability (Fig 3), reduced the clearance of damaged mitochondria, and increased the mitochondrial membrane potential and their resistance to oxidative stress in H9C2 cells (Fig 4-5).
4. Per the reviewer's request, we provided more details regarding the anoxia methodology (lines 155-156 in "Materials & Methods"), and- as listed above, two references pointing that oxygen deprivation can indeed lead to MitoSOX accumulation (27, 28).

We declare that the funders had no role in study design, data collection and analysis, decision to publish, or preparation of the manuscript.

We hope that our revised manuscript will now be found suitable for publication in PLOS ONE.

Sincerely,

Dr. Michal Entin-Meer and Prof. Gad Keren
